# Does outcome expectancy predict outcomes in online depression prevention? Secondary analysis of randomised‐controlled trials

**DOI:** 10.1111/hex.13951

**Published:** 2023-12-28

**Authors:** Janika Thielecke, Paula Kuper, David Ebert, Pim Cuijpers, Filip Smit, Heleen Riper, Dirk Lehr, Claudia Buntrock

**Affiliations:** ^1^ Professorship of Psychology and Digital Mental Health Care, Department of Sports and Health Sciences Technical University of Munich Munich Germany; ^2^ Department of Clinical Psychology and Psychotherapy, Institute of Psychology Friedrich‐Alexander ‐University Erlangen‐Nürnberg Erlangen Germany; ^3^ The Netherlands Organization for Applied Scientific Research (TNO) Leiden The Netherlands; ^4^ Institute of Social Medicine and Health Systems Research, Faculty of Medicine Otto‐von‐Guericke University Magdeburg Magdeburg Germany; ^5^ Department of Clinical, Neuro and Developmental Psychology VU University Amsterdam The Netherlands; ^6^ Amsterdam Public Health Amsterdam University Medical Centers Amsterdam The Netherlands; ^7^ Department of Mental Health and Prevention Trimbos Institute, Netherlands Institute of Mental Health and Addiction Utrecht The Netherlands; ^8^ Department of Epidemiology and Biostatistics University Medical Center Amsterdam msterdam The Netherlands; ^9^ Department of Psychiatry VU University Medical Center Amsterdam The Netherlands; ^10^ Department of Health Psychology and Applied Biological Psychology Leuphana University Luneburg Lüneburg Germany

**Keywords:** CBT, depression, expectancy, online intervention, prediction, prevention, secondary analyses

## Abstract

**Background:**

Evidence shows that online interventions could prevent depression. However, to improve the effectiveness of preventive online interventions in individuals with subthreshold depression, it is worthwhile to study factors influencing intervention outcomes. Outcome expectancy has been shown to predict treatment outcomes in psychotherapy for depression. However, little is known about whether this also applies to depression prevention. The aim of this study was to investigate the role of participants' outcome expectancy in an online depression prevention intervention.

**Methods:**

A secondary data analysis was conducted using data from two randomised‐controlled trials (*N* = 304). Multilevel modelling was used to explore the effect of outcome expectancy on depressive symptoms and close‐to‐symptom‐free status postintervention (6–7 weeks) and at follow‐up (3–6 months). In a subsample (*n* = 102), Cox regression was applied to assess the effect on depression onset within 12 months. Explorative analyses included baseline characteristics as possible moderators. Outcome expectancy did not predict posttreatment outcomes or the onset of depression.

**Results:**

Small effects were observed at follow‐up for depressive symptoms (*β* = −.39, 95% confidence interval [CI]: [−0.75, −0.03], *p* = .032, *p*_adjusted_ = .130) and close‐to‐symptom‐free status (relative risk = 1.06, 95% CI: [1.01, 1.11], *p* = .013, *p*_adjusted_ = 0.064), but statistical significance was not maintained when controlling for multiple testing. Moderator analyses indicated that expectancy could be more influential for females and individuals with higher initial symptom severity.

**Conclusion:**

More thoroughly designed, predictive studies targeting outcome expectancy are necessary to assess the full impact of the construct for effective depression prevention.

**Patient or Public Contribution:**

This secondary analysis did not involve patients, service users, care‐givers, people with lived experience or members of the public. However, the findings incorporate the expectations of participants using the preventive online intervention, and these exploratory findings may inform the future involvement of participants in the design of indicated depression prevention interventions for adults.

**Clinical Trial Registration:**

Original studies: DRKS00004709, DRKS00005973; secondary analysis: osf.io/9xj6a.

## 1 INTRODUCTION

Subthreshold depression (sD) is highly prevalent, with estimates ranging from 1.4% to 17.2% in community samples^1^ and associated with poor quality of life,^2^, ^3^ a high risk of developing the major depressive disorder (MDD),^4^, ^5^ increased mortality^6^ and the use of healthcare services,^7^ as well as substantial economic costs.^8^ Individuals are considered to suffer from sD if they show clinically relevant depressive symptoms but standard diagnostic criteria for a MDD are not yet met.^3^ sD can be defined categorically by meeting some but not all criteria for a MDD diagnosis or dimensionally by scoring above a certain cut‐off level on validated self‐rated depression scales.^9^

The importance of preventive interventions aimed at individuals with sD (i.e., indicated prevention) is emphasised by the fact that nearly all individuals who develop a MDD are assumed to have first gone through a phase of sD.^10^ Meta‐analytic evidence shows that face‐to‐face preventive psychological interventions can reduce the incidence of depression by about 20% (relative risk [RR] = 0.81, 95% CI confidence interval [CI]: [0.72, 0.91]).^11^ Though clearly effective, psychological face‐to‐face interventions do not reach the majority of people who could benefit from them.^12^ Online interventions have the potential to increase access to preventive services.

Online interventions target cognitive, affective and behavioural changes; are typically based on evidence‐based face‐to‐face interventions; and require active engagement from participants through the completion of online and offline assignments.^13^ Discussed benefits of online interventions include flexible use, comparably low costs and the ability to reach a wide range of users.^12^, ^14^, ^15^ A recent individual participant data (IPD) meta‐analysis on guided and unguided online interventions showed that they can be effective in improving depressive symptom severity (*d* = −0.39, 95% CI: [−0.25, −0.53]) and in reducing the incidence of MDD in individuals with sD (hazard ratio [HR] = 0.72, 95% CI: [0.58–0.89]).^16^ However, to further increase the effectiveness of preventive online interventions for depression, it is important to investigate factors that predict differential treatment outcome.^17^

Patient expectancies of treatment outcome—that is, their beliefs about whether treatment will lead to an improvement in health status^18^, ^19^—are discussed as a common factor in psychotherapy^20^, ^21^ and are meta‐analytically associated with psychotherapy outcomes (*r* = .12–.18).^18^, ^19^ Depression‐specific studies have shown the predictive value of a positive outcome expectancy in individual face‐to‐face treatment of *β* = −.35 on 16‐week follow‐up depression scores.^22^ In group CBT (cognitive behavioural therapy), an indirect effect of outcome expectancy of *β* = −1.29 [95% CI: −2.93, −0.16] on depression scores at posttreatment after 10 weeks was observed that was mediated by mid‐treatment working alliance.^23^ Outcome expectancy has attracted attention as a way to maximise treatment outcomes.^24^, ^25^, ^26^, ^27^

In online interventions, outcome expectancy has mainly been investigated in terms of the acceptability and uptake of various internet health services^13^, ^28^, ^29^ and less in its persistent effects on the outcome. A recent meta‐analysis comparing the effect of outcome expectancy in face‐to‐face and online interventions suggested similar predictive effects in both modalities in the treatment of anxiety, tinnitus and chronic pain.^30^ Evidence for an association with depression outcomes in guided and unguided online interventions is inconclusive, with three studies supporting outcome expectancy as being predictive for treatment outcome,^31^, ^32^, ^33^ whereas four studies did not find that association.^34^, ^35^, ^36^, ^37^ Another study reported an effect of outcome expectancy fully mediated by working alliance as reported by patients.^38^

For preventive online interventions, evidence is scant and indecisive, with outcome expectancy showing correlations with a reduction in anxiety symptoms^39^ but not with posttreatment obsessive–compulsive disorder symptoms.^40^ To the best of our knowledge, no study has investigated outcome expectancy for a preventive online intervention for depression. The aim of this study was thus to explore the predictive role of outcome expectancy in a preventive online intervention for adults with sD. We hypothesised that outcome expectancy predicted depressive symptom severity (research question 1 [RQ1]) and close‐to‐symptom‐free status (RQ2) at posttreatment and follow‐up and depression onset within 12 months (RQ3). In exploratory analyses, we examined the moderating effects of baseline symptom severity, age and sex (exploratory RQ1–3).

## 2 METHODS

### 2.1 Study design and participants

**Figure 1:**
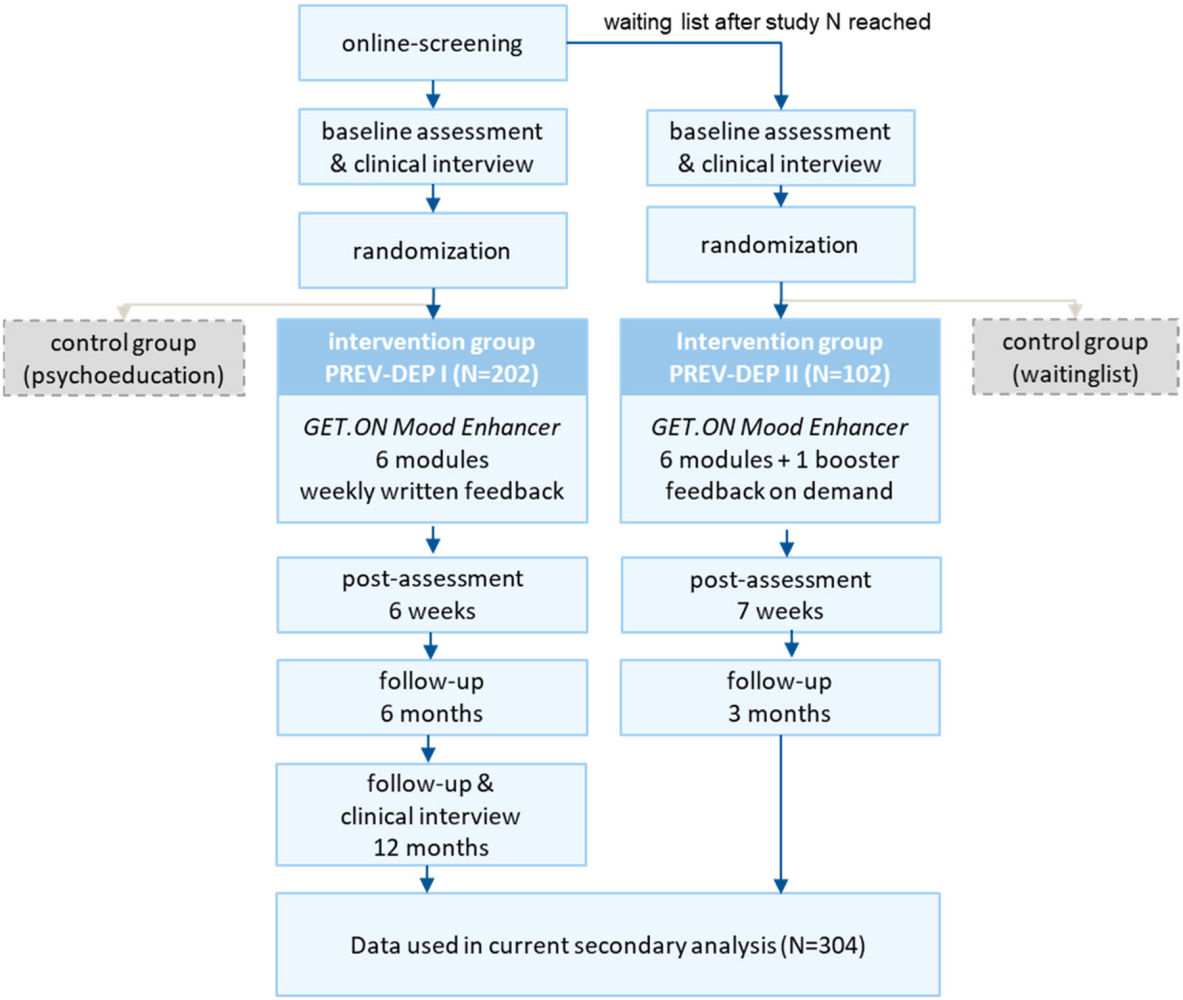
Participant flow in the original study and inclusion in the secondary analyses. PREV‐DEP I and PREV‐DEP II are the study acronyms used for the original studies.^41^, ^42^

Secondary analyses were conducted based on combined data from the intervention groups of two randomised‐controlled trials (RCTs) (*N* = 304) that evaluated the effectiveness of the same online intervention (GET.ON Mood Enhancer Prevention). Earlier publications from these trials reported effects of the intervention on depressive symptoms^41^, ^42^ and the progression to a major depressive episode during a 12‐month follow‐up period.^43^ The first study (PREV‐DEP I, *N* = 406) compared the online intervention with enhanced treatment‐as‐usual (i.e., online psychoeducation) within a 12‐month follow‐up period,^44^ while the second study (PREV‐DEP II, *N* = 204) compared the intervention with a wait‐list control condition within a 3‐month follow‐up period.^42^ Figure 1 shows an overview of the original studies' design. The studies were approved by the ethics committee of the Philipps University Marburg (2012‐35K, PREV‐DEP I) and the Leuphana University Lueneburg (Ebert201404_Depr, PREV‐DEP II), respectively, and registered in the German Clinical Trial Register under DRKS00004709 and DRKS00005973. These secondary analyses of pseudonymized data were registered on OSF (https://doi.org/10.17605/OSF.IO/9XJ6A) prior to before data analysis.

Participants were mainly recruited via a large German statutory health insurance company (BARMER) by announcing the studies in the members' magazine. The studies were also announced in newspaper articles, on‐air media and related websites. The open recruitment strategy, which mimicked the way in which people are likely to be recruited for online preventive interventions, provided ecological validity to the study sample. Applicants self‐identifying with a depressed mood and who (a) screened positive for subthreshold depressive symptoms (Centre for Epidemiological Studies Depression Scale [CES‐D] ≥ 16),^45^ (b) were 18 years of age or older, (c) had internet access, (d) were neither currently in psychotherapy (e) nor in the past 6 months (f) or on a waiting list for psychotherapy and (g) showed no notable suicidal risk (Becks Depression Inventory [BDI item 9] ≤ 1)^46^ were scheduled for a semi‐structured clinical interview (SCID) conducted via telephone by trainees in psychotherapy to assess final eligibility. Those who met DSM‐IV criteria for (a) a major depressive episode, (b) bipolar disorder or (c) psychotic disorder and (d) with a history of a MDD in the past 6 months were excluded. As trials were pragmatic, the use of antidepressant medication was allowed as part of treatment‐as‐usual if participants took a stable dose for at least 4 weeks before study participation.

### 2.2 Intervention

The online intervention consisted of six 30‐min modules. The session duration could vary between users. In PREV‐DEP II, participants were offered an optional seventh module as a booster session 4 weeks after completion of the intervention. Each module integrated texts, practical exercises and testimonials and interactive components, such as audio‐guided relaxation exercises and videos that explain theoretical frameworks. Participants in both studies were advised to complete two modules per week if possible, but at least one. This led to a flexible duration of intervention completion, ranging from 3 to 6 weeks. The intervention was based on psychoeducation, behaviour therapy (BT) and problem‐solving therapy (PST). BT underscores the importance of daily pleasurable activity scheduling. PST involves a structured approach (i.e., a six‐step procedure) to problem‐solving. The programme concludes with elective modules in the final three modules, covering sleep hygiene, relaxation techniques and coping with worry. An emphasis was placed on transfer tasks, that is, homework assignments, designed to embed acquired strategies into participants' daily routines. Optionally, participants could opt for standardised text messages as reminders (e.g., brief relaxation techniques). While participants in the PREV‐DEP I were supported by an online coach who provided written individual feedback after each completed module, participants in PREV‐DEP II received feedback only upon request. In both studies, feedback focused on helping participants complete the exercises, and no therapeutic advice was provided.

### 2.3 Measurements

#### 2.3.1 Outcome expectancy

Outcome expectancy was assessed at baseline before the start of the intervention with the respective items from the Credibility and Expectancy Questionnaire (CEQ).^47^ The CEQ version used in PREV‐DEP I & II was self‐translated into German and not validated in this form. The wording was adapted to specify ‘online‐training’ as the intervention and ‘depressive symptoms’ as the outcome. The CEQ expectancy subscale included one item on how participants think and two items on how they *feel* about the effect that the intervention will have on their depressive symptoms. Items were rated on a scale from 1 to 9, leading to a composite score for expectancy ranging from 3 (low expectancy) to 27 (high expectancy). The original CEQ demonstrated good psychometric properties, with high internal consistency and high test–retest reliability.^47^ Cronbach's *α* in the combined data was *α* = .87.

#### 2.3.2 Depressive symptom severity

Depressive symptom severity was measured using the German version of the CES‐D.^48^ The CES‐D is a self‐reporting scale consisting of 20 items, each scored from 0 to 3, resulting in a total score from 0 to 60, with a higher score indicating more severe depressive symptoms. The psychometric properties of the CES‐D are well established.^48^ Cronbach's *α* in combined data was *α* = .82 at baseline, *α* = .89 at posttreatment and *α* = .90 at follow‐up. Close‐to‐symptom‐free status was defined by a CES‐D score <16.

#### 2.3.3 Onset of major depressive episode

Time to onset of a major depressive episode over a 12‐month follow‐up was only assessed in PREV‐DEP I. DSM‐IV criteria were assessed via telephone‐administered SCID at the 6‐ and 12‐month follow‐up, covering the period from the previous assessment.^49^, ^50^ To reduce potential recall bias, time to onset of MDD was assessed as accurately as possible using the Life Chart method.^51^ Diagnostic interviews were conducted by psychologists trained in delivering the SCID. The *κ* coefficient for inter‐rater agreement for a diagnosis of a depressive episode was 0.77 (based on data from 12% of the participants), indicating substantial agreement.^52^

### 2.4 Prognostic factors

Age, sex and baseline depressive symptom severity have been repeatedly identified as predictors of outcome in online interventions for depression treatment.^31^, ^35^, ^37^, ^53^ These baseline characteristics were included in the analyses to assess an adjusted effect of expectancy.

### 2.5 Data analysis

For combined data, ‘posttreatment’ refers to the first assessment after intervention completion and ‘follow‐up’ defines the second assessment after three (PREV‐DEP II) and 6 months (PREV‐DEP I), respectively. For each hypothesis, separate regression models were specified in R.^54^ Significance levels for the five effect estimates of expectancy on depressive outcomes were adjusted for multiple testing using the Bonferroni–Holm method.^55^ Effects of expectancy were reported with the appropriate estimates and CIs. *R* ^2^ was reported as a measure of overall model fit. The full model specifications are given in Supporting Information S1: Online Resource 1.

### 2.6 Depressive symptom severity (H1 and H2)

To account for the nesting of participants in trials, RQ1 is answered using multilevel models. First, a one‐stage IPD approach^56^ was used to investigate the predictive effect of expectancy on depressive symptom severity. The general recommendations for one‐step IPD analyses from Riley et al.^56^ were followed, i.e., specifying a stratified intercept, a random slope of expectancy and stratified prognostic variables (age, sex, baseline CES‐D), each centred by their trial means. Second, given the small number of included studies (*k* = 2), we followed the proposal by Chung et al.^57^ to use a ‘pseudo‐Bayesian’ approach in the multilevel models. Between‐study heterogeneity was highly plausible, given the differences in session count, guidance and assessment points in the trials, but the small number of random‐effects levels (trials) may have led to improperly estimated heterogeneity variances at zero. Using a weakly informative gamma prior with a shape parameter of 1.5 and a rate parameter of 0.05 allowed for an approximate maximum a posteriori estimate.^58^ The prior, implemented with the ‘blme’ package,^59^ helped to avoid boundary‐fit problems while remaining largely uninformative itself.

Models 1 and 2 were defined as linear mixed‐effects models predicting depressive symptoms postintervention (H1) and at follow‐up (H2) based on expectancy.

Depression onset (H3) is answered based on PREV‐DEP I data alone and thus did not need to account for the nesting of data within studies. Model 3 was specified as a right‐censored Cox regression using the ‘survival’^60^ and ‘survminer’ package.^61^ Continuous baseline characteristics were mean‐centred. HRs with their 95% CIs were reported. In addition to Nagelkerke's pseudo *R*² calculated using the ‘psfmi’^62^ package, concordance as a more adequate goodness‐of‐fit measure was given.^60^

### 2.7 Close‐to‐symptom‐free status (H4 and H5)

To answer RQ3, the same approach as described above for RQ1 was used. Close‐to‐symptom‐free status was predicted from expectancy in models 4 (posttreatment) and 5 (follow‐up) using generalised linear‐effects models with a clog‐log link function to retrieve RRs. Participants with CES‐D < 16 at baseline, despite inclusion criteria were excluded from this analysis. Model 5 included close‐to‐symptom‐free status at posttreatment as an additional covariate, which was stratified and centred. The prior rate was adjusted to 0.01 in model 5 to reduce convergence problems. However, both models 4 and 5 showed convergence problems in one of the 50 imputed sets.

#### 2.7.1 Explorative analyses

As exploratory analyses, an interaction term of expectancy and each baseline characteristic (age, sex, baseline symptom severity) was entered into separate linear mixed‐effects models examining differential depressive symptoms while additionally controlling for baseline characteristics (comparable to H1). Continuous candidate moderators were centred by trial‐specific means. Contrary to the preregistration, we did not include trial means of candidate moderators as a level 2 predictor as recommended by Riley et al.^56^ because of multicollinearity with the trial‐specific intercepts. However, as suggested, we stratified and centred all parameters outside the interaction term including expectancy in order to avoid amalgamation of within‐ and across‐trial information.^56^

### 2.8 Missing data

Analyses were conducted according to the intention‐to‐treat principle. Multiple imputation by chained equations (fully conditional specification) was applied using the R packages ‘mice’^63^ and ‘miceadds,’^64^ assuming that data were missing at random. A total of *m* = 50 imputation sets were generated and visually inspected for plausibility (Supporting Information S1: Online Resource 2). Model parameters were estimated in all data sets and combined according to Rubin's rules.^65^, ^66^

A multilevel imputation model was used to account for the nested structure of the data (participants in trials). Continuous data were imputed using ‘2l.pan’,^67^ a special case of a multivariate linear mixed‐effects model for panel data already included in ‘mice’. Due to convergence problems, cluster means of the covariates could not be included in the prediction matrix. For congeniality of imputation and analysis models, the ‘blme’^59^ functionality was called up within ‘mice’ to apply a weakly informative gamma‐prior with a shape parameter of 1.5 and a rate of 0.05.

For RQ3, no imputation was needed since baseline data were complete and data for depression onset were right‐censored, with observation time set to 0 or 26 weeks in cases of dropout that occurred after baseline or the 6‐month follow‐up, respectively (*n* = 40).

## 3 RESULTS

### 3.1 Participants

In PREV‐DEP I, participant data were missing in 12% of cases (25/202) at posttreatment, and in 25% (51/202) and 36% (72/202) at the 6‐ and 12‐month follow‐up due to study dropout.^41^, ^43^ In PREV‐DEP II, dropout was 21% (21/102) and 29% (30/102), respectively, at posttreatment and the 3‐month follow‐up.^42^ Study dropout (*r* = .03, *T*(302) = 0.7, *p* = .5) and number of completed sessions (*r* = .04, *T*(302) = 0.8, *p* = .4) were not related to baseline expectancy. Participants across both trials (Table 1) were predominantly female (*n* = 230, 76%), highly educated (*n* = 195, 64%), in a relationship (*n* = 166, 55%) and on average 45 years old (SD = 11.8). Expectancy ranged from low (min = 4) to high (max = 27), with *M* = 16.7 (SD = 5.0).

**Table 1:** Baseline characteristics of participants in both trials and the combined sample.

	Total (*N* = 304)^a^		PREV‐DEP I^b^ (*N* = 202)		PREV‐DEP II^c^ (*N* = 102)	
	*N*	%	*N*	%	*N*	%
CES‐D sum score (M, SD)	26.4	7.4	26.3	7.9	26.7	6.5
CEQ expectancy (M, SD)^a^	16.7	5.0	16.8	5.1	16.4	4.8
Age (M, SD)	45.4	11.8	45.7	11.9	44.7	11.7
Gender						
Male	73	24	53	26	20	20
Female	231	76	149	74	82	80
Relationship						
Single	89	29	62	31	27	27
Married or cohabiting	167	55	102	50	65	64
Divorced or separated	46	15	37	18	9	9
Widowed	3	1	2	1	1	1
Ethnicity						
White	244	80	165	81	79	78
Black	1	0	1	1	0	0
Not reported	60	20	37	18	23	23
Level of education						
Low (primary)^d^	7	2	5	3	2	2
Middle (secondary)^e^	49	16	33	16	16	16
High (A‐level or higher)^f^	248	82	164	81	84	82
Employment status						
Employed	260	86	170	84	90	88
Unemployed or seeking work	6	2	4	2	2	2
On sick‐leave	3	1	3	2	0	0
Nonworking	36	12	26	12	10	10
Income in Euro^g^						
Low (<10,000)	25	8	16	8	9	9
Middle (10–60,000)	215	71	145	72	70	69
High (>60,000)	40	13	26	13	14	14
Not reported	27	9	18	9	9	9
Previous						
Psychotherapy	170	56	88	44	82	40
Health training^h^	74	24	51	25	23^a^	23
Use of antidepressants	57	19	50	25	7	7

Abbreviations: CES‐D, Centre for Epidemiologic Studies Depression Scale; CEQ, Credibility and Expectancy Questionnaire.

^a^ New data added in the current work.

^b^ Adapted from Buntrock et al.^41^

^c^ Adapted from Ebert et al.^15^

^d^ Primary education indicates elementary school.

^e^ Secondary education indicates high school.

^f^ Indicates A‐level examinations (‘Abitur’) or above (university degree).

^g^ Yearly gross income (€1.00 ≈ US $1.13 at the time of the original study)

^h^ Preventive interventions as offered by German statutory health insurance companies (e.g., stress management, smoking cessation, healthy diet).

### 3.2 Depressive symptom severity (H1 and H2)

No predictive effect of expectancy on depressive symptom severity at posttreatment was observed (*β* = −.30, 95% CI: [−0.73, 0.13], *p*_adjusted_ = .352, *R*² = .21, *N* = 304). Higher expectancy at baseline indicated lower depressive symptom scores at follow‐up (*β* = −.39, 95% CI: [−0.75, −0.03] *N* = 304). This effect did not remain significant when controlling for multiple testing (*p* = .032, *p*_adjusted_ = .130, *R*² = .26).

### 3.3 Depression onset (H3)

As reported elsewhere,^43^ from the *n* = 202 participants in PREV‐DEP I, *n* = 55 (27.2%) individuals experienced onset of depression within the follow‐up period. No predictive effect of expectancy on depression onset was found (HR = 0.97, 95% CI: [0.93, 1.02], *p*_adjusted_ = .346). The proportional hazard assumptions were met according to the scaled Schoenfeld residual test (global *χ*²[4] = 0.10 *p* = 1.00). The model was overall significant based on the Likelihood ratio test (*χ* ^2^[4] = 16.25, *p* = .003) but did not explain the data well (concordance = 0.66, SE = 0.04, Nagelkerke's pseudo *R*² = .08).

### 3.4 Close‐to‐symptom‐free status (H4 and H5)

For analyses of close‐to‐symptom‐free status, participants already close‐to‐symptom‐free status at baseline were excluded (*n* = 16), resulting in *n* = 290. No predictive effect of expectancy on close‐to‐symptom‐free status at posttreatment was found (RR = 1.04, 95% CI: [0.99, 1.08], *p*_adjusted_ = .326, *R*² = .11). Comparably to H1/H2, a positive effect of expectancy on reaching close‐to‐symptom‐free status at follow‐up was observed (RR = 1.06, 95% CI: [1.01, 1.11]) but statistical significance did not remain after adjusting for multiple testing (*p* = .013, *p*_adjusted_ = .064, *R*² = .29).

## 4 MODERATION ANALYSES (EXPLORATORY H1–H3)

While higher baseline expectancy was potentially associated with lower depressive symptoms at posttreatment, sex (*β* = .46, 95% CI: [0.03, 0.90], *p* = .038, *R*² = .22, *N* = 304) and baseline severity (*β* = −.03, 95% CI: [−0.05, 0.001], *p* = .041, *R*² = .22, *N* = 304) significantly moderated this association, but not age (*β* = .01, 95% CI: [−0.01, 0.02], *p* = .270, *R*² = 0.21, *N* = 304). With each one‐point increase from the trial‐specific mean in expectancy, depressive symptom severity at posttreatment was reduced by an additional 0.46 points for females. Each additional one‐point increase from the trial‐specific mean in initial depressive symptom severity increased the effect of expectancy on depressive symptom severity by 0.03 points. No moderating effect was observed at follow‐up assessment (Supporting Information S1: Online Resource 3).

## 5 DISCUSSION

In this secondary analysis, we explored the effect of expectancy in an online intervention for indicated depression prevention on different depressive outcomes at posttreatment and follow‐up. No predictive effects of expectancy on posttreatment depressive symptom severity, close‐to‐symptom‐free status or time to depression onset within 12 months could be observed. At follow‐up, small effects were found on depressive symptom severity (*β* = −.39, 95% CI: [−0.75, −0.03], *p* = .032, *p*_adjusted_ = .130) and close‐to‐symptom‐free status (RR = 1.06, 95% CI: [1.01–1.11], *p* = .013, *p*_adjusted_ = .064), but significance was lost after adjusting for multiple testing. Exploratory analyses suggested that female sex and higher depressive symptom severity increased the effect of expectancy on symptom severity at posttreatment.

Our findings are in line with other studies that did not find an effect of expectancy on depression outcomes directly after use of an online intervention.^34^, ^35^, ^36^, ^37^ However, given the possible effects at follow‐up, overall evidence remains inconclusive. Our observation that expectancy might be more relevant in later follow‐ups than in posttreatment is similar to findings reported by de Graaf et al.,^32^ who observed a predictive effect of expectancy on reliably changed depression scores after 9 months, but not after 3 months. Indeed, studies that did not find an effect of expectancy all had observation times under 10 weeks.^34^, ^35^, ^36^, ^37^ Zagorscak et al.^38^ suggest that early expectancy predicts mid‐treatment task and goal agreement, which then leads to symptom improvement.^38^ This might explain a delayed effect on depressive outcomes in self‐help‐oriented interventions, where identification with tasks and goals might be crucial for the transfer into everyday life.

Studies that found a predictive effect of expectancy shared common methods, namely, that they used a validated questionnaire (i.e., CEQ) and either did not include a randomisation process and assessed expectancy after session one or two^31^, ^33^ or they assessed expectancy after randomisation.^32^ We also used the CEQ expectancy scale; however, we assessed expectancy before randomisation. Not knowing whether they had immediate access to the online intervention might have influenced participants' expectancy ratings.

To our knowledge, our exploratory moderation analyses are the first of their kind in online interventions. However, these findings are contrary to what has been found in in‐person therapy, where age was observed to be a moderator, but not sex.^19^ Higher baseline depressive symptom severity has previously been reported to correlate with lower expectancy.^19^, ^68^, ^69^ This would indicate that individuals with more severe symptoms at baseline are a prime target group when trying to enhance expectancy before preventive interventions and that possible sex‐specific responses should be considered.

The large heterogeneity in existing studies' definitions, instruments, measurement times of expectancy and outcome assessments and intervention characteristics restricts the comparability of study results in all healthcare fields.^70^, ^71^ Outcome expectancy has been transferred as a common factor from in‐person psychotherapy to online intervention but little research is done on how comparable treatment mechanisms are across prevention versus treatment or in‐person versus online interventions.^72^ The baseline value of expectancy, being only slightly above the middle of the scale (range: 3–27, *M* = 16.67, SD = 5.00), raises a question about whether participants had distinct expectations about changes in depressive symptoms or if the tendency towards the midpoint represents uncertainty of what to expect from an (online) prevention intervention.^73^ The novelty of online preventive interventions and the assessment of expectancies before randomisation could contribute to this uncertainty, assuming that individuals generally have an idea of the psychotherapy effects. More (qualitative) research is needed to understand what individuals expect when signing up for a preventive online intervention for depression and what kind of information participants used to derive their expectations when encountering novel interventions.

### 5.1 Implications for research and practice

Some evidence already exists that outcome expectancy can increase the intention to use online interventions.^29^, ^74^ The results of our study suggest that it could be worthwhile to assess and foster outcome expectancy before the start of a preventive online intervention for depression, considering that it is as an easily assessable, influenceable and amenable characteristic before and during an intervention.^27^, ^75^ However, more thoroughly designed, predictive studies targeting expectancy are required to ascertain the full impact of expectancy on the effectiveness of depression prevention.

Such studies should, first and foremost, apply validated measurement instruments of expectancy and control for confounding effects (e.g., credibility or working alliance).^18^, ^19^, ^47^ To the best of our knowledge, no comparative studies have been conducted to assess how expectancy is influenced by the underlying disorder. This might, however, be crucial when applying the concept to depression prevention and treatment, given that depression is associated with generally more pessimistic expectations.^76^ In addition, as a common factor in in‐person psychotherapy, the association between expectancy and symptoms of depression posttreatment is discussed as being (partly) mediated through therapeutic alliance, a mechanism also found in guided online interventions.^38^ However, definitions and operationalizations of therapeutic alliance in online interventions may differ from those used in face‐to‐face psychotherapy research and as a consequence, need to be assessed with specific measures for online interventions.^77^

Expectancy should be discussed with regard to the amount of guidance provided. For studies examining social anxiety interventions, Nordgreen et al.^78^ summarised that expectancy was a predictor for symptom reduction in the unguided but not in the guided intervention arm. However, similar conclusions could not be drawn for depression interventions.^38^ Studying outcome expectancy in relation to the intensity of guidance (e.g., unguided, adherence‐focused, guidance on demand, guided) might help to maximise individual benefits.

Future studies should also systematically evaluate when outcome expectancy is best assessed to be able to draw conclusions about its impact. Constantino et al.^19^ combined studies that used pretreatment and early treatment expectancies in their meta‐analysis, assuming that there was no relevant difference in expectancy. This assumption is challenged by the findings in online interventions that only studies assessing expectancy after exposure to the intervention observed an association with the outcome. Formerly, having some experience with the intervention was considered to be only important for reliable credibility measurements, but not for outcome expectancy.^18^

Finally, it would be prudent to conduct longitudinal studies to understand how initial outcome expectancy emerges, how it changes and interacts as more experience with the intervention is gained and how this affects its role as a predictor of treatment outcome. These studies would also need to take into account the familiarity of the sample with online interventions and prevention, as well as previous intervention experience,^79^ information available before treatment decision^80^ and level of human support. To test whether expectancy plays a specific role in online interventions, studies directly comparing online and face‐to‐face preventive offers are warranted. This information could assist in designing interventions or components to foster expectancy starting from the initial help‐seeking impulse and throughout the intervention.

If methodologically sound studies establish outcome expectancy as a predictor of depressive outcomes in online interventions, further research should investigate whether manipulating outcome expectancy before and during an intervention (e.g., providing a strong intervention rationale, managing unrealistic expectations, providing a nontechnical overview of the efficacy of online interventions) indeed results in greater effects (e.g., reduced risk for depression onset), taking into account participants' characteristics (e.g., initial symptom severity, sex).

### 5.2 Limitations

Our study includes secondary analyses of RCT data that were not originally designed to examine expectancy. Thus, some limitations need to be considered when interpreting the results: First, the German version of the CEQ used in the studies was self‐translated, not validated in a German sample and adapted in wording. This might have influenced the validity of the instrument.^81^ Also, no further attitudinal or motivational attributes were accessed, which could help with the interpretation of the CEQ baseline scores.

Second, even though we combined two trials, power might have been insufficient to detect small predictive effects of expectancy, especially because the question focusing on depression onset was present in only one study. The sample size also did not allow us to consider study‐level characteristics such as guidance as potential moderators, more complex associations like nonlinear trends or moderating effects by multiple variables, which should be considered in future research. The sample size was partly reduced as we did not include participants in the control conditions, given the different operationalization and given that expectancy was only assessed with regard to the online intervention.

Third, although age, sex and baseline depressive symptom severity have been included in the analyses to assess an adjusted effect of expectancy, other prognostic indicators for MDD could not be included (e.g., history of MDD, chronic medical conditions).

Fourth, previous experiences in healthcare are of interest to better understand outcome expectancies. Even though data on experience with previous health trainings and psychotherapy were assessed in the original studies, these data could not be used because we lacked information on whether these experiences were perceived as positive or negative.^19^

## 6 CONCLUSION

In this secondary analysis of two RCTs for indicated online depression prevention, we could not find a predictive effect of outcome expectancy on depressive symptoms at posttreatments or on depression onset. Models predicting follow‐up depression scores and moderation analyses appear promising, but more research is needed to assess the potential impact of including more participant‐focused characteristics such as expectancy to enhance effectiveness in preventive online interventions for depression.

## AUTHOR CONTRIBUTIONS

**Janika Thielecke**: Conceptualisation; writing—original draft; writing—review and editing; methodology; formal analysis; visualisation. **Paula Kuper**: Methodology; formal analysis; conceptualisation; writing—review and editing. **David Ebert**: Writing—review and editing; conceptualisation. **Pim Cuijpers**: writing—review and editing; conceptualisation. **Filip Smit**: Writing—review and editing; conceptualisation. **Heleen Riper**: Writing—review and editing; conceptualisation. **Dirk Lehr**: Writing—review and editing; conceptualisation. **Claudia Buntrock**: Writing—review and editing; investigation; methodology; conceptualisation; project administration; data curation; supervision; resources; writing—original draft.

## CONFLICT OF INTEREST STATEMENT

David Ebert is a stakeholder of the GET.ON Institute/HelloBetter, which aims to implement scientific findings related to digital health interventions into routine care. David Ebert has served as a consultant to/on the scientific advisory boards of Sanofi, Novartis, Minddistrict, Lantern, Schoen Kliniken, Ideamed and German health insurance companies (BARMER, Techniker Krankenkasse) and a number of federal chambers for psychotherapy. All other authors report no conflicts of interest.

## ETHICS STATEMENT

The original studies were performed in line with the principles of the Declaration of Helsinki. Approval was granted by the Ethics Committee of the Philipps University Marburg (2012‐35 K, PREV‐DEP I) and the Leuphana University Lueneburg (Ebert201404_Depr, PREV‐DEP II). No additional approval for the secondary analysis of the pseudonomized data were needed. All participants signed a written informed consent before being enroled in the studies.

## Supporting information

Supporting information.

## Data Availability

Access to the final pseudonymized trial data set that support the findings of this study can be provided to fellow researchers upon request from the last author, depending on to be specified data security and data exchange regulation agreements. The analyses scripts can be accessed via OSF: osf.io/9xj6a.
