# Supplementary material for: Does outcome expectancy predict outcomes in online depression prevention? Secondary analysis of randomised‐controlled trials
Source: Health Expect. 2023 Dec 28;27(1):e13951. doi: 10.1111/hex.13951 (PMC10753640; doi:10.1111/hex.13951)
Supplement: Supplementary file 1 — Supporting information. [file HEX-27-e13951-s001.docx]

**Online Resource 1 – Full model specifications**

***RQ1: Are outcome expectancy and depression symptom severity at post and follow-up related?***

***Hypothesis 1***

H0(1) = Outcome expectancy at baseline does not predict depressive symptomatology at post-treatment.
H1(1) = Outcome expectancy at baseline does predict depressive symptomatology at post-treatment.

*Model specification:*

with(implist,expr= blmer(cesd_1 ~ 0+ factor(trial)+ trial1_cesd_0_c+trial2_cesd_0_c +trial1_age_c+trial2_age_c+ trial1_sex_c + trial2_sex_c +ceq_exp + (0 + ceq_exp | trial),

control = lmerControl(optimize= "Nelder_Mead"),

cov.prior = trial~"gamma"(shape=1.5, rate=0.05) ))

*Full model:*

| **term** | **estimate** | **std.error** | **statistic** | **df** | **p.value** | **lower 95%-CI** | **upper 95%-CI** |
| --- | --- | --- | --- | --- | --- | --- | --- |
| factor(trial)1 | 24.6310 | 3.1526 | 7.8130 | 160.0 | 0.0000 | 18.4049 | 30.8570 |
| factor(trial)2 | 21.0965 | 2.0241 | 10.4230 | 244.0 | 0.0000 | 17.1096 | 25.0830 |
| trial1_cesd_0_c | 0.5350 | 0.1420 | 3.7680 | 186.0 | 0.0002 | 0.2549 | 0.8150 |
| trial2_cesd_0_c | 0.4669 | 0.0782 | 5.9710 | 235.0 | 0.0000 | 0.3129 | 0.6210 |
| trial1_age_c | 0.0105 | 0.0781 | 0.1340 | 225.0 | 0.8934 | -0.1435 | 0.1640 |
| trial2_age_c | 0.0888 | 0.0530 | 1.6740 | 208.0 | 0.0956 | -0.0158 | 0.1930 |
| trial1_sex_c | -1.9171 | 2.2667 | -0.8460 | 205.0 | 0.3987 | -6.3862 | 2.5520 |
| trial2_sex_c | 2.5911 | 1.4078 | 1.8400 | 226.0 | 0.0670 | -0.1831 | 5.3650 |
| ceq_exp | -0.2981 | 0.2197 | -1.3570 | 272.0 | 0.1760 | -0.7307 | 0.1340 |

***Hypothesis 2***

H0(2) = Outcome expectancy at baseline does not predict depressive symptomatology at follow-up assessment.
H1(2) = Outcome expectancy at baseline does predict depressive symptomatology at follow-up assessment.

*Model specification:*

with(implist,expr= blmer(cesd_2 ~ 0+ factor(trial)+ trial1_cesd_0_c+trial2_cesd_0_c +trial1_age_c+trial2_age_c+ trial1_sex_c + trial2_sex_c +ceq_exp + (0 + ceq_exp | trial),

control = lmerControl(optimize= "Nelder_Mead"),

cov.prior = trial~"gamma"(shape=1.5, rate=0.05) ))

*Full model:*

| **term** | **estimate** | **std.error** | **statistic** | **df** | **p.value** | **lower 95%-CI** | **upper 95%-CI** |
| --- | --- | --- | --- | --- | --- | --- | --- |
| factor(trial)1 | 24.3340 | 3.1270 | 7.7820 | 141.0 | 0.0000 | 18.1523 | 30.5158 |
| factor(trial)2 | 23.8453 | 2.1099 | 11.3010 | 202.0 | 0.0000 | 19.6850 | 28.0057 |
| trial1_cesd_0_c | 0.6002 | 0.1500 | 4.0010 | 156.0 | 0.0001 | 0.3039 | 0.8965 |
| trial2_cesd_0_c | 0.5146 | 0.0828 | 6.2170 | 196.0 | 0.0000 | 0.3514 | 0.6779 |
| trial1_age_c | 0.0255 | 0.0880 | 0.2900 | 143.0 | 0.7723 | -0.1484 | 0.1994 |
| trial2_age_c | 0.1643 | 0.0579 | 2.8350 | 152.0 | 0.0052 | 0.0498 | 0.2787 |
| trial1_sex_c | -0.9581 | 2.3131 | -0.4140 | 199.0 | 0.6792 | -5.5195 | 3.6033 |
| trial2_sex_c | 3.8596 | 1.4880 | 2.5940 | 189.0 | 0.0102 | 0.9244 | 6.7948 |
| ceq_exp | -0.3913 | 0.1819 | -2.1520 | 244.0 | 0.0324 | -0.7495 | -0.0331 |

## *RQ2: Are outcome expectancy and time to depression onset within a 12-month follow-up period related?*

***Hypothesis 3***

H0(3) = In the subset of participants with clinical interviews, outcome expectancy at baseline does not predict time to depression onset at 12-months follow-up assessment.

H1(3) = In the subset of participants with clinical interviews, outcome expectancy at baseline does predict time to depression onset at 12-months follow-up assessment

*Model specification:*

Surv(fu.time, dep.status, type = "right") ~ ceq.exp + sex + age.c + cesd.0.c

*Full model:*

|  | **coef** | **exp(coef)** | **se(coef)** | **z** | **p.value** | **lower 95%-CI** | **upper 95%-CI** |
| --- | --- | --- | --- | --- | --- | --- | --- |
| ceq.exp | -0.028576 | 0.9718 | 0.024588 | -1.162 | 0.2452 | 0.9261 | 1.02 |
| sex1 | 0.155361 | 1.1681 | 0.302467 | 0.514 | 0.6075 | 0.6457 | 2.113 |
| age.c | 0.007878 | 1.0079 | 0.010941 | 0.72 | 0.4715 | 0.9865 | 1.03 |
| cesd.0.c | 0.068768 | 1.0712 | 0.017678 | 3.89 | 0.0001 | 1.0347 | 1.109 |

## *RQ3: Are outcome expectancy and “close-to-symptom-free”-status at post and follow-up related?*

***Hypothesis 4***

H0(4) = Outcome expectancy at baseline does not predict “close-to-symptom-free”-status at post-treatment.
H1(4) = Outcome expectancy at baseline does predict “close-to-symptom-free”-status at post-treatment.

*Model specification:*

with(implist, expr= bglmer(symptom_free_1 ~ 0 + factor(trial+trial1_cesd_0_c+ trial2_cesd_0_c+trial1_age_c+trial2_age_c+ + trial1_sex_c + trial2_sex_c + ceq_exp + (0 + ceq_exp | trial),

family=binomial(link= "cloglog"),

control = glmerControl(optimizer= "Nelder_Mead"),

cov.prior = trial~"gamma"(shape=1.5, rate=0.05)))

*Full model:*

| **term** | **estimate** | **std.error** | **statistic** | **df** | **p.value** | **RR** | **RR** | **RR** |
| --- | --- | --- | --- | --- | --- | --- | --- | --- |
|  |  |  |  |  |  |  | **CI.lower** | **CI.upper** |
| factor(trial)1 | -1.4615 | 0.4311 | -3.3903 | 219.9 | 0.0008 | 0.2319 | 0.0992 | 0.5423 |
| factor(trial)2 | -1.2606 | 0.4109 | -3.0681 | 225.9 | 0.0024 | 0.2835 | 0.1262 | 0.6370 |
| trial1_cesd_0_c | -0.0904 | 0.0336 | -2.6937 | 202.4 | 0.0077 | 0.9136 | 0.8551 | 0.9761 |
| trial2_cesd_0_c | -0.0661 | 0.0189 | -3.4976 | 254.8 | 0.0006 | 0.9360 | 0.9018 | 0.9715 |
| trial1_age_c | -0.0009 | 0.0152 | -0.0571 | 248.2 | 0.9545 | 0.9991 | 0.9697 | 1.0295 |
| trial2_age_c | -0.0154 | 0.0109 | -1.4191 | 233 | 0.1572 | 0.9847 | 0.9638 | 1.0060 |
| trial1_sex_c | 0.3696 | 0.4136 | 0.8935 | 218.3 | 0.3726 | 1.4471 | 0.6404 | 3.2698 |
| trial2_sex_c | -0.5263 | 0.3175 | -1.6579 | 213.7 | 0.0988 | 0.5908 | 0.3160 | 1.1045 |
| ceq_exp | 0.0363 | 0.0225 | 1.6113 | 225.3 | 0.1085 | 1.0369 | 0.9920 | 1.0840 |

***Hypothesis 5***

H0(5) = Outcome expectancy at baseline does not predict “close-to-symptom-free”-status at follow-up assessment.
H1(5) = Outcome expectancy at baseline does predict “close-to-symptom-free”-status at follow-up assessment.

*Model specification:*

fit.blmer <- with(implist,expr= bglmer(symptom_free_2 ~ 0 + trial + trial1_cesd_0_c +trial2_cesd_0_c +trial1_age_c+trial2_age_c+ + trial1_sex_c + trial2_sex_c + ceq_exp + trial1_symptom_free_1_c + trial2_symptom_free_1_c + (0 + ceq_exp | trial),

family=binomial(link= "cloglog"),

control = glmerControl(optimize= "Nelder_Mead"),

cov.prior = trial~"gamma"(shape=1.5, rate=0.01)))

*Full model:*

| **term** | **estimate** | **std.error** | **statistic** | **df** | **p.value** | **RR** | **RR CI.lower** | **RR CI.upper** |
| --- | --- | --- | --- | --- | --- | --- | --- | --- |
| trial1 | -1.3466 | 0.4490 | -2.9992 | 157.0 | 0.0031 | 0.2601 | 0.1072 | 0.6314 |
| trial2 | -1.7490 | 0.4514 | -3.8747 | 159.7 | 0.0002 | 0.1740 | 0.0713 | 0.4242 |
| trial1_cesd_0_c | -0.0435 | 0.0297 | -1.4644 | 182.3 | 0.1448 | 0.9575 | 0.9030 | 1.0152 |
| trial2_cesd_0_c | -0.0550 | 0.0233 | -2.3584 | 181.1 | 0.0194 | 0.9464 | 0.9038 | 0.9910 |
| trial1_age_c | -0.0069 | 0.0139 | -0.4970 | 171.7 | 0.6198 | 0.9931 | 0.9661 | 1.0208 |
| trial2_age_c | -0.0145 | 0.0114 | -1.2722 | 182.6 | 0.2049 | 0.9856 | 0.9638 | 1.0080 |
| trial1_sex_c | -0.1083 | 0.4119 | -0.2629 | 184.3 | 0.7929 | 0.8974 | 0.3981 | 2.0225 |
| trial2_sex_c | -0.2840 | 0.3396 | -0.8362 | 186.1 | 0.4041 | 0.7528 | 0.3852 | 1.4711 |
| ceq_exp | 0.0599 | 0.0238 | 2.5178 | 164.9 | 0.0128 | 1.0618 | 1.0130 | 1.1128 |
| trial1_symptom_free_1_c | -1.0023 | 0.3616 | -2.7720 | 182.5 | 0.0061 | 0.3670 | 0.1798 | 0.7491 |
| trial2_symptom_free_1_c | -1.4666 | 0.2976 | -4.9277 | 169.0 | 0.0000 | 0.2307 | 0.1282 | 0.4152 |

**Supplement Table 1**

*p-values adjustment for multiple testing in hypotheses 1-5 using the Bonferroni-Holm method*

| **hypothesis** | **p_unadjusted_** | **p_adjusted_** |
| --- | --- | --- |
| H5: CEQ -> Close-to-symptom-free-status follow-up | 0.0128 | 0.0638 |
| H2: CEQ -> CES-D follow-up | 0.0324 | 0.1296 |
| H4: CEQ -> Close-to-symptom-free-status post | 0.1085 | 0.3255 |
| H1: CEQ -> CES-D post | 0.1760 | 0.3520 |
| H3: CEQ -> Depression onset | 0.2452 | 0.3520 |

*Note.* Table sorted according to original unadjusted p-values.

**Exploratory RQ1: Is the relation between outcome expectancy and depression outcome at post-treatment moderated by baseline characteristics?**

***Exploratory Hypothesis 1***

Exploratory H0(1): Age does not moderate the effect of outcome expectancy on depressive symptoms at post-treatment.
Exploratory H1(1): Age moderates the effect of outcome expectancy on depressive symptoms at post-treatment.

*Model specification:*

ws.interaction.age <-ceq_exp_c*age_c

with(implist, expr= blmer(cesd_1 ~ 0 + trial + trial1_cesd_0_c +trial2_cesd_0_c +trial1_age_c+trial2_age_c + trial1_sex_c+trial2_sex_c+ trial1_ceq_exp_c + trial2_ceq_exp_c + ws.interaction.age + (0 + ceq_exp |trial),
control = lmerControl(optimize= "Nelder_Mead"),
cov.prior = trial~"gamma"(shape=1.5, rate=0.05)))

*Full model:*

| **term** | **estimate** | **std.error** | **statistic** | **df** | **p.value** | **lower 95%-CI** | **upper 95%-CI** |
| --- | --- | --- | --- | --- | --- | --- | --- |
| trial1 | 25.1966 | 3.4203 | 7.3667 | 172.6 | 0.0000 | 18.4455 | 31.9477 |
| trial2 | 20.8763 | 2.0971 | 9.9546 | 246.7 | 0.0000 | 16.7457 | 25.0069 |
| trial1_cesd_0_c | 0.5317 | 0.1422 | 3.7394 | 184.7 | 0.0002 | 0.2512 | 0.8123 |
| trial2_cesd_0_c | 0.4643 | 0.0784 | 5.9250 | 232.8 | 0.0000 | 0.3099 | 0.6186 |
| trial1_age_c | -0.1216 | 0.1489 | -0.8169 | 236.7 | 0.4148 | -0.4150 | 0.1717 |
| trial2_age_c | -0.0523 | 0.1396 | -0.3746 | 239.6 | 0.7083 | -0.3273 | 0.2227 |
| trial1_sex_c | -1.9389 | 2.2687 | -0.8546 | 203.7 | 0.3938 | -6.4120 | 2.5342 |
| trial2_sex_c | 2.6589 | 1.4100 | 1.8857 | 224.3 | 0.0606 | -0.1197 | 5.4375 |
| trial1_ceq_exp_c | -0.4296 | 80.9893 | -0.0053 | 289.0 | 0.9958 | -159.8333 | 158.9741 |
| trial2_ceq_exp_c | -0.1998 | 80.9891 | -0.0025 | 289.0 | 0.9980 | -159.6031 | 159.2036 |
| ws.interaction.age | 0.0083 | 0.0075 | 1.1068 | 260.9 | 0.2694 | -0.0065 | 0.0231 |
|  |  |  |  |  |  |  |  |

***Exploratory Hypothesis 2***

Exploratory H0(2): Sex does not moderate the effect of outcome expectancy on depressive symptoms at post-treatment.
Exploratory H1(2): Sex moderates the effect of outcome expectancy on depressive symptoms at post-treatment.

*Model specification:*

ws.interaction.sex <-ceq_exp_c*sex

with(implist, expr= blmer(cesd_1 ~ 0 + trial + trial1_cesd_0_c +trial2_cesd_0_c +trial1_age_c+trial2_age_c + trial1_sex_c+trial2_sex_c + trial1_ceq_exp_c + trial2_ceq_exp_c + ws.interaction.sex + (0 + ceq_exp |trial),

control = lmerControl(optimize= "Nelder_Mead"),

cov.prior = trial~"gamma"(shape=1.5, rate=0.05)))

*Full model:*

| **term** | **estimate** | **std.error** | **statistic** | **df** | **p.value** | **lower 95%-CI** | **upper 95%-CI** |
| --- | --- | --- | --- | --- | --- | --- | --- |
| trial1 | 25.0347 | 3.4051 | 7.3521 | 171.9 | 0.0000 | 18.3135 | 31.7559 |
| trial2 | 21.3247 | 2.1051 | 10.1299 | 243.7 | 0.0000 | 17.1781 | 25.4712 |
| trial1_cesd_0_c | 0.5336 | 0.1414 | 3.7746 | 184.4 | 0.0002 | 0.2547 | 0.8125 |
| trial2_cesd_0_c | 0.4556 | 0.0779 | 5.8456 | 234.2 | 0.0000 | 0.3020 | 0.6092 |
| trial1_age_c | 0.0121 | 0.0784 | 0.1544 | 223.1 | 0.8775 | -0.1425 | 0.1667 |
| trial2_age_c | 0.0938 | 0.0529 | 1.7711 | 205.2 | 0.0780 | -0.0106 | 0.1981 |
| trial1_sex_c | -9.6631 | 4.4398 | -2.1765 | 223.2 | 0.0306 | -18.4124 | -0.9138 |
| trial2_sex_c | -5.0249 | 3.9122 | -1.2844 | 251.8 | 0.2002 | -12.7296 | 2.6798 |
| trial1_ceq_exp_c | -0.9658 | 80.5190 | -0.0120 | 289.0 | 0.9904 | -159.4437 | 157.5122 |
| trial2_ceq_exp_c | -0.8011 | 80.5189 | -0.0099 | 289.0 | 0.9921 | -159.2790 | 157.6767 |
| ws.interaction.sex | 0.4608 | 0.2212 | 2.0832 | 253.4 | 0.0382 | 0.0252 | 0.8965 |

***Exploratory Hypothesis 3***

Exploratory H0(3): Baseline depressive symptoms does not moderate the effect of outcome expectancy on depressive symptoms at post-treatment.
Exploratory H1(3): Baseline depressive symptoms moderates the effect of outcome expectancy on depressive symptoms at post-treatment.

*Model specification:*

ws.interaction.cesd_0 <-ceq_exp_c*cesd_0_c

with(implist, expr= blmer(cesd_1 ~ 0 + trial + trial1_cesd_0_c +trial2_cesd_0_c +trial1_age_c+trial2_age_c + trial1_sex_c+trial2_sex_c + trial1_ceq_exp_c +trial2_ceq_exp_c + ws.interaction.cesd_0 + (0 + ceq_exp |trial),

control = lmerControl(optimize= "Nelder_Mead"),

cov.prior = trial~"gamma"(shape=1.5, rate=0.05)))

*Full model:*

| **term** | **estimate** | **std.error** | **statistic** | **df** | **p.value** | **lower 95%-CI** | **upper 95%-CI** |
| --- | --- | --- | --- | --- | --- | --- | --- |
| trial1 | 25.2781 | 3.4021 | 7.4302 | 171.9 | 0.0000 | 18.5629 | 31.9932 |
| trial2 | 20.8803 | 2.0879 | 10.0005 | 245.0 | 0.0000 | 16.7677 | 24.9929 |
| trial1_cesd_0_c | 0.9434 | 0.2425 | 3.8909 | 216.4 | 0.0001 | 0.4655 | 1.4214 |
| trial2_cesd_0_c | 0.8994 | 0.2286 | 3.9350 | 211.4 | 0.0001 | 0.4488 | 1.3500 |
| trial1_age_c | 0.0188 | 0.0785 | 0.2391 | 222.6 | 0.8112 | -0.1359 | 0.1734 |
| trial2_age_c | 0.0920 | 0.0530 | 1.7359 | 203.3 | 0.0841 | -0.0125 | 0.1965 |
| trial1_sex_c | -1.9212 | 2.2574 | -0.8511 | 202.5 | 0.3957 | -6.3723 | 2.5299 |
| trial2_sex_c | 2.8279 | 1.4029 | 2.0157 | 225.5 | 0.0450 | 0.0633 | 5.5924 |
| trial1_ceq_exp_c | -0.4256 | 80.4910 | -0.0053 | 289.0 | 0.9958 | -158.8486 | 157.9973 |
| trial2_ceq_exp_c | -0.1962 | 80.4909 | -0.0024 | 289.0 | 0.9981 | -158.6188 | 158.2265 |
| ws.interaction.cesd_0 | -0.0252 | 0.0123 | -2.0575 | 228.2 | 0.0408 | -0.0494 | -0.0011 |

**Online Resource 2 – Additional information multiple imputation**

**Figure 1**

Predictor matrix indicating which variables were used in the imputation model.

**
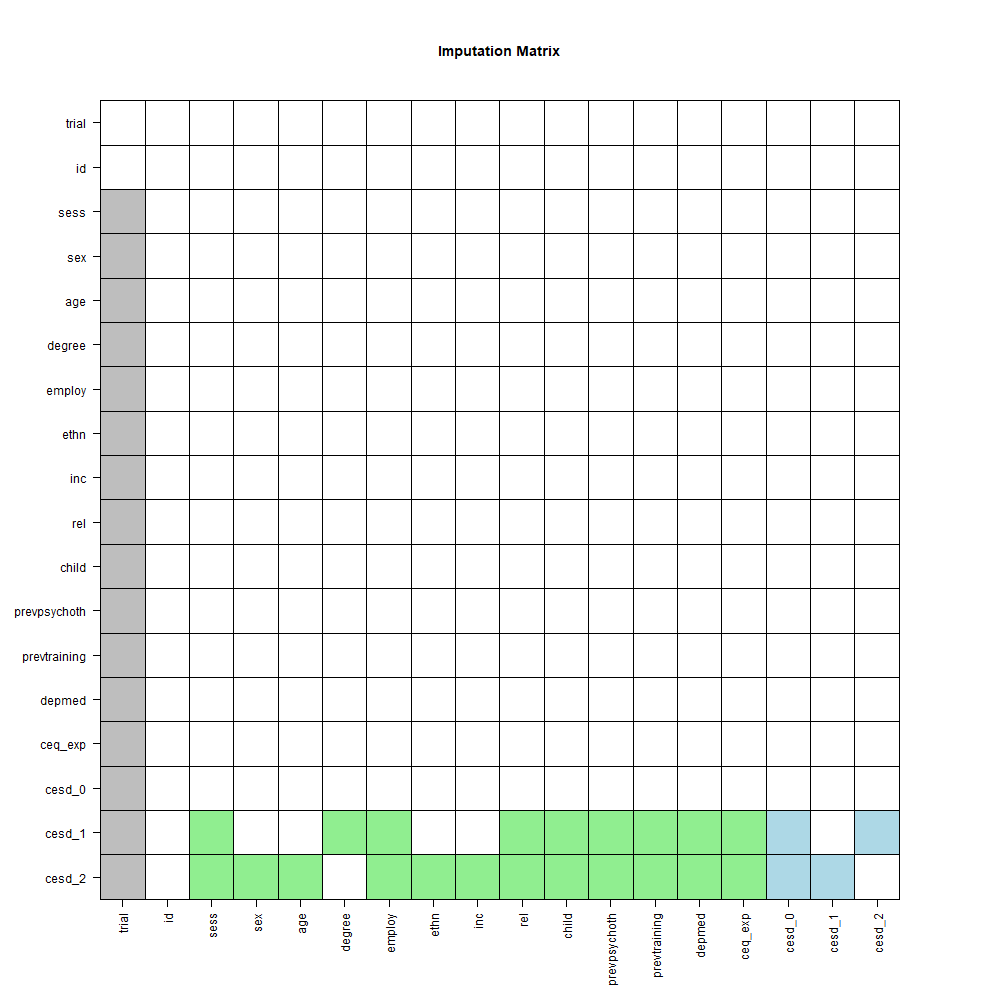
**

Note. Values in the rows (CES-D at post-treatment and follow-up) are predicted by the variables in the columns. The trial variable (grey) was used as the cluster variable. A multilevel model with participants being nested in trials was used to predict missing outcomes. Depressive symptom severity (CES-D at baseline, post intervention and follow-up) was modeled as a fixed and random effect (blue). Other variables were modeled as simple linear predictors (green).

**Figure 2**

Trace plots depicting the mean and standard deviations of the imputed outcome values (depression scores measured with CES-D) at post treatment and follow-up per iteration and imputation set.

**
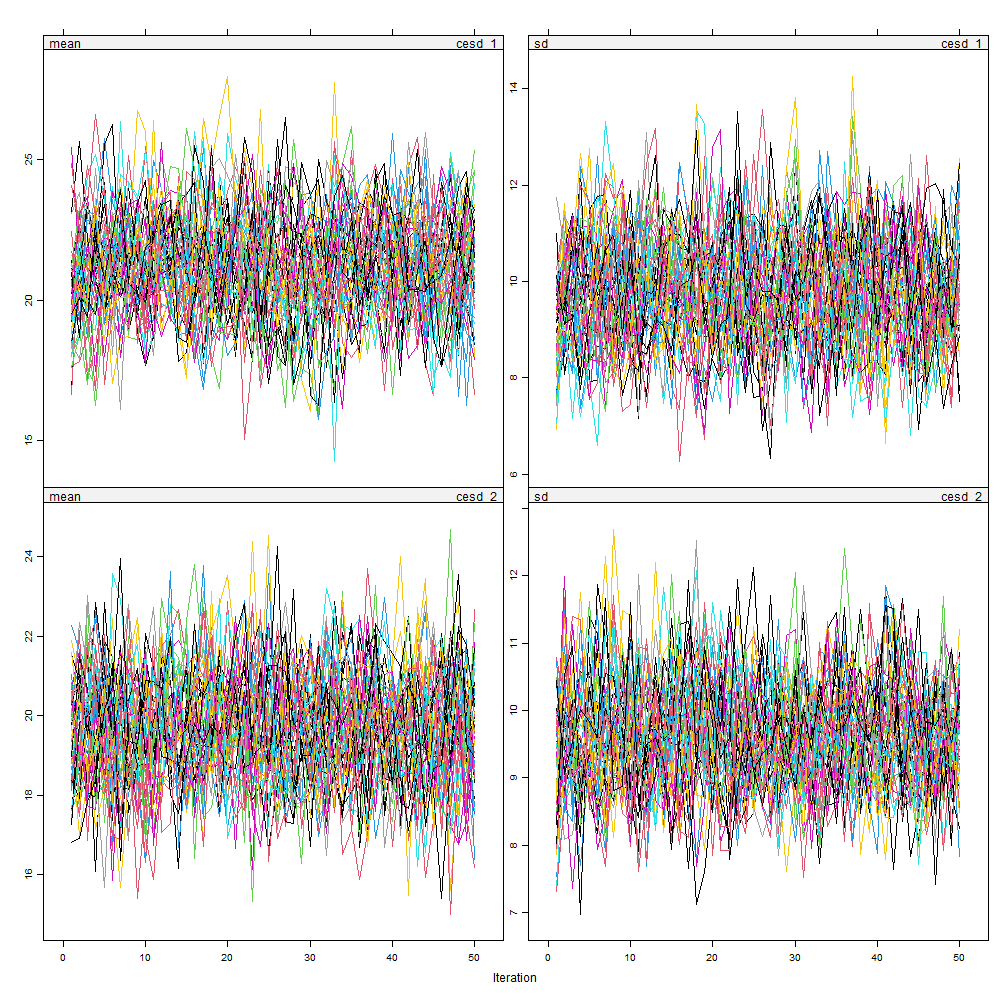
**

**Figure 3**

Density plots for the outcome variables (depression scores measured with CES-D) in the original data (blue) and imputed data (red; for each imputed dataset).

**
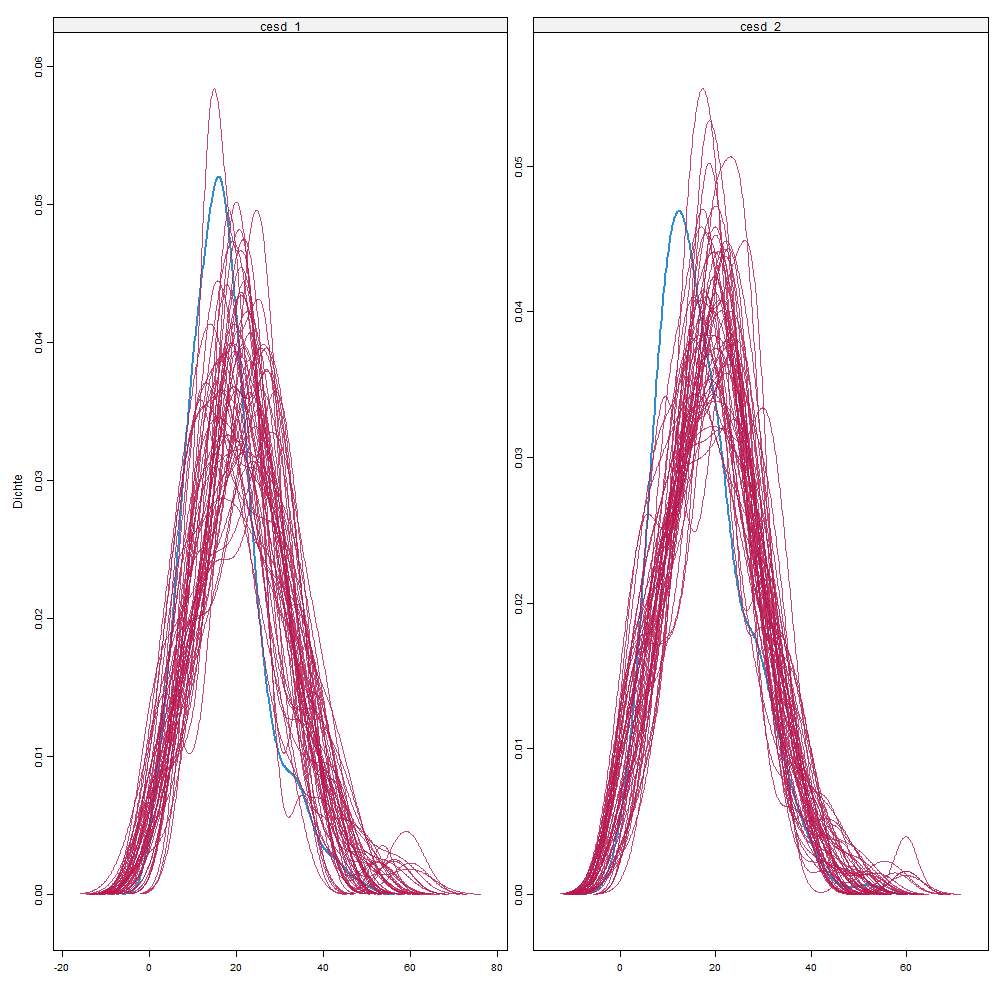
**

**Online Resource 3 – Additional analysis: Moderation at Follow-Up**

**Additional Exploratory RQ: Is the relation between outcome expectancy and depression outcome at follow-up moderated by baseline characteristics?**

***Exploratory Hypothesis 4***

Exploratory H0(4): Age does not moderate the effect of outcome expectancy on depressive symptoms at follow-up assessment..
Exploratory H1(4): Age moderates the effect of outcome expectancy on depressive symptoms at follow-up assessment..

*Model specification:*

ws.interaction.age <-ceq_exp_c*age_c

with(implist, expr= blmer(cesd_2 ~ 0 + trial + trial1_cesd_0_c +trial2_cesd_0_c +trial1_age_c+trial2_age_c + trial1_sex_c+trial2_sex_c+ trial1_ceq_exp_c + trial2_ceq_exp_c + ws.interaction.age + (0 + ceq_exp |trial),
control = lmerControl(optimize= "Nelder_Mead"),
cov.prior = trial~"gamma"(shape=1.5, rate=0.05)))

*Full model:*

| **term** | **estimate** | **std.error** | **statistic** | **df** | **p.value** | **lower 95%-CI** | **upper 95%-CI** |
| --- | --- | --- | --- | --- | --- | --- | --- |
| trial1 | 24.6997 | 3.6450 | 6.7764 | 141.9 | 0.0000 | 17.4942 | 31.9051 |
| trial2 | 23.6968 | 2.2172 | 10.6877 | 210.1 | 0.0000 | 19.3260 | 28.0677 |
| trial1_cesd_0_c | 0.5992 | 0.1505 | 3.9824 | 155.7 | 0.0001 | 0.3020 | 0.8964 |
| trial2_cesd_0_c | 0.5136 | 0.0830 | 6.1858 | 195.3 | 0.0000 | 0.3499 | 0.6774 |
| trial1_age_c | -0.0238 | 0.1678 | -0.1417 | 153.8 | 0.8875 | -0.3553 | 0.3077 |
| trial2_age_c | 0.1102 | 0.1497 | 0.7362 | 191.4 | 0.4625 | -0.1851 | 0.4055 |
| trial1_sex_c | -0.9614 | 2.3210 | -0.4142 | 197.4 | 0.6792 | -5.5384 | 3.6157 |
| trial2_sex_c | 3.8885 | 1.4923 | 2.6057 | 188.8 | 0.0099 | 0.9448 | 6.8322 |
| trial1_ceq_exp_c | -0.4509 | 82.2459 | -0.0055 | 289.0 | 0.9956 | -162.3279 | 161.4262 |
| trial2_ceq_exp_c | -0.3498 | 82.2458 | -0.0043 | 289.0 | 0.9966 | -162.2264 | 161.5269 |
| ws.interaction.age | 0.0032 | 0.0081 | 0.3921 | 207.1 | 0.6954 | -0.0128 | 0.0191 |
|  |  |  |  |  |  |  |  |

***Exploratory Hypothesis 5***

Exploratory H0(5): Sex does not moderate the effect of outcome expectancy on depressive symptoms at follow-up assessment.
Exploratory H1(5): Sex moderates the effect of outcome expectancy on depressive symptoms at follow-up assessment.

*Model specification:*

ws.interaction.sex <-ceq_exp_c*sex

with(implist, expr= blmer(cesd_2 ~ 0 + trial + trial1_cesd_0_c +trial2_cesd_0_c +trial1_age_c+trial2_age_c + trial1_sex_c+trial2_sex_c + trial1_ceq_exp_c + trial2_ceq_exp_c + ws.interaction.sex + (0 + ceq_exp |trial),

control = lmerControl(optimize= "Nelder_Mead"),

cov.prior = trial~"gamma"(shape=1.5, rate=0.05)))

*Full model:*

| **term** | **estimate** | **std.error** | **statistic** | **df** | **p.value** | **lower 95%-CI** | **upper 95%-CI** |
| --- | --- | --- | --- | --- | --- | --- | --- |
| trial1 | 24.6771 | 3.6373 | 6.7845 | 143.2 | 0.0000 | 17.4875 | 31.8668 |
| trial2 | 23.7766 | 2.2463 | 10.5847 | 204.0 | 0.0000 | 19.3477 | 28.2056 |
| trial1_cesd_0_c | 0.6003 | 0.1503 | 3.9941 | 156.0 | 0.0001 | 0.3034 | 0.8972 |
| trial2_cesd_0_c | 0.5124 | 0.0832 | 6.1561 | 194.8 | 0.0000 | 0.3482 | 0.6765 |
| trial1_age_c | 0.0279 | 0.0897 | 0.3108 | 140.2 | 0.7564 | -0.1494 | 0.2051 |
| trial2_age_c | 0.1649 | 0.0582 | 2.8342 | 151.0 | 0.0052 | 0.0500 | 0.2799 |
| trial1_sex_c | -2.4711 | 4.9809 | -0.4961 | 150.9 | 0.6205 | -12.3123 | 7.3702 |
| trial2_sex_c | 2.3686 | 4.4227 | 0.5355 | 166.1 | 0.5930 | -6.3633 | 11.1004 |
| trial1_ceq_exp_c | -0.5551 | 82.2290 | -0.0068 | 289.0 | 0.9946 | -162.3988 | 161.2885 |
| trial2_ceq_exp_c | -0.4669 | 82.2289 | -0.0057 | 289.0 | 0.9955 | -162.3103 | 161.3766 |
| ws.interaction.sex | 0.0905 | 0.2512 | 0.3604 | 164.4 | 0.7190 | -0.4054 | 0.5865 |

***Exploratory Hypothesis 6***

Exploratory H0(6): Baseline depressive symptoms does not moderate the effect of outcome expectancy on depressive symptoms at follow-up assessment.
Exploratory H1(6): Baseline depressive symptoms moderates the effect of outcome expectancy on depressive symptoms at follow-up assessment.

*Model specification:*

ws.interaction.cesd_0 <-ceq_exp_c*cesd_0_c

with(implist, expr= blmer(cesd_2 ~ 0 + trial + trial1_cesd_0_c +trial2_cesd_0_c +trial1_age_c+trial2_age_c + trial1_sex_c+trial2_sex_c + trial1_ceq_exp_c +trial2_ceq_exp_c + ws.interaction.cesd_0 + (0 + ceq_exp |trial),

control = lmerControl(optimize= "Nelder_Mead"),

cov.prior = trial~"gamma"(shape=1.5, rate=0.05)))

*Full model:*

| **term** | **estimate** | **std.error** | **statistic** | **df** | **p.value** | **lower 95%-CI** | **upper 95%-CI** |
| --- | --- | --- | --- | --- | --- | --- | --- |
| trial1 | 24.7355 | 3.6373 | 6.8006 | 141.9 | 0.0000 | 17.5452 | 31.9257 |
| trial2 | 23.7056 | 2.2155 | 10.7001 | 208.9 | 0.0000 | 19.3381 | 28.0732 |
| trial1_cesd_0_c | 0.8174 | 0.2581 | 3.1675 | 180.9 | 0.0018 | 0.3082 | 1.3266 |
| trial2_cesd_0_c | 0.7445 | 0.2428 | 3.0660 | 177.9 | 0.0025 | 0.2653 | 1.2236 |
| trial1_age_c | 0.0303 | 0.0898 | 0.3379 | 138.2 | 0.7360 | -0.1473 | 0.2080 |
| trial2_age_c | 0.1659 | 0.0581 | 2.8541 | 150.1 | 0.0049 | 0.0510 | 0.2807 |
| trial1_sex_c | -0.9583 | 2.3153 | -0.4139 | 197.7 | 0.6794 | -5.5241 | 3.6075 |
| trial2_sex_c | 3.9866 | 1.4886 | 2.6781 | 191.1 | 0.0080 | 1.0504 | 6.9228 |
| trial1_ceq_exp_c | -0.4496 | 82.0792 | -0.0055 | 289.0 | 0.9956 | -161.9983 | 161.0992 |
| trial2_ceq_exp_c | -0.3487 | 82.0790 | -0.0042 | 289.0 | 0.9966 | -161.8971 | 161.1997 |
| ws.interaction.cesd_0 | -0.0134 | 0.0131 | -1.0258 | 189.8 | 0.3063 | -0.0392 | 0.0124 |
